# Primary Isolated Lacrimal Gland Amyloidosis: A Case Report and Review of the Literature

**DOI:** 10.7759/cureus.6258

**Published:** 2019-11-29

**Authors:** William Evans, Barrett Thompson, Stephen C Dryden, Caroline Awh, Brian Fowler

**Affiliations:** 1 Ophthalmology, University of Tennessee Health Science Center, Memphis, USA

**Keywords:** amyloidosis, lacrimal gland, orbital amyloidosis

## Abstract

We report a case of a 42-year-old woman who presented with a one-year history of a painless right orbital mass and right upper lid ptosis. Magnetic resonance imaging (MRI) revealed a superotemporal right orbital mass involving the lacrimal gland, and subsequent tissue biopsy and histopathologic evaluation was consistent with amyloidosis. An otherwise negative workup by hematology/oncology confirmed a diagnosis of primary isolated lacrimal gland amyloidosis. To the best of our knowledge, this is the first documented case of isolated lacrimal gland amyloidosis without immunoglobulin (Ig) light chain restriction on in situ hybridization testing with a report of MRI findings. In addition, this is the second reported case of the disease without Ig light chain restriction on immunohistochemistry staining, and it is the third case with reported MRI results.

## Introduction

Amyloidosis is a group of disorders characterized by impaired protein metabolism with subsequent extracellular deposition of fibrils [1]. This disease is often secondary to either genetic mutations or systemic inflammatory disorders. In 88% of cases, amyloidosis is systemic [2]. In contrast to patients with systemic disease, 12% of patients with localized disease have a favorable prognosis with only 1% progressing to systemic light chain amyloidosis. Among patients with isolated disease, lacrimal gland involvement is rare and with only 19 cases reported to date, there is little known about this disease entity [3,4]. In this group of patients, immunohistochemical findings have infrequently been discussed and typically show light chain restriction [4-7]. Additionally, magnetic resonance imaging (MRI) results have only been reported in two other cases [3,6]. In this report, we document the first reported case of primary isolated lacrimal gland amyloidosis without immunoglobulin (Ig) light chain restriction on in situ hybridization testing with a report of MRI findings.

## Case presentation

**Figure 1:**
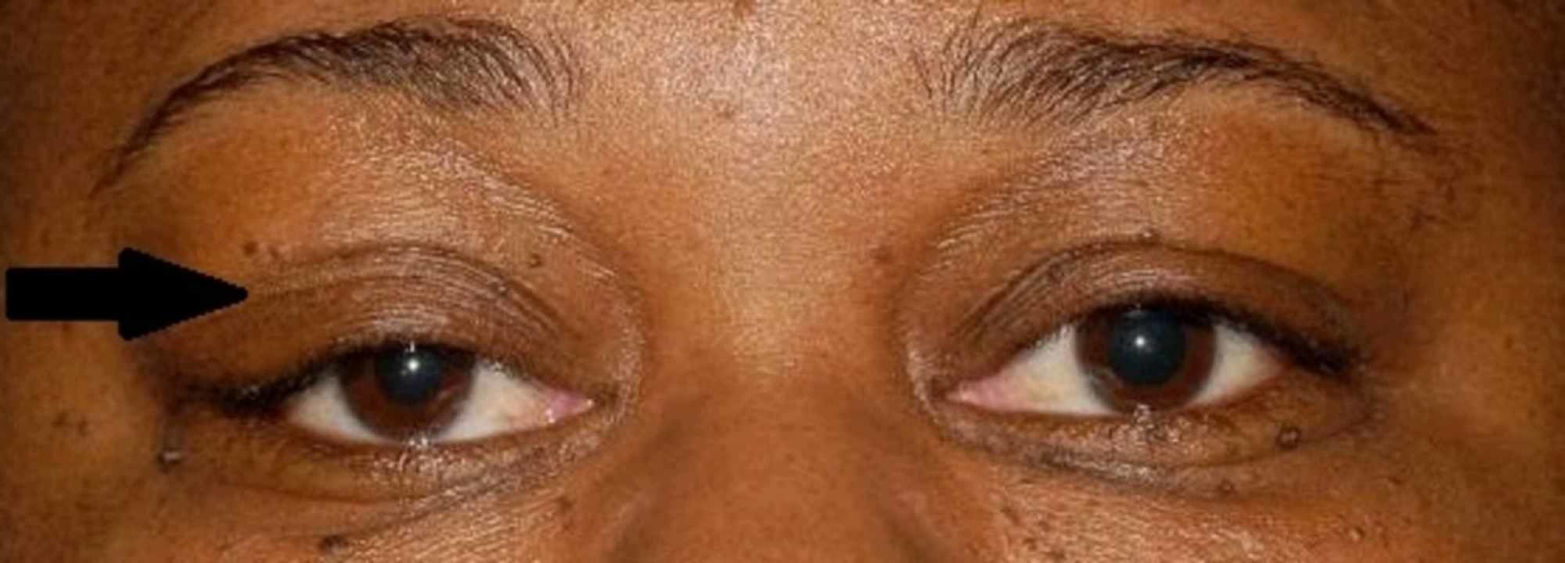
External photograph External photo showing a ptosis of the right upper eyelid and fullness of the right orbit.

**Figure 2:**
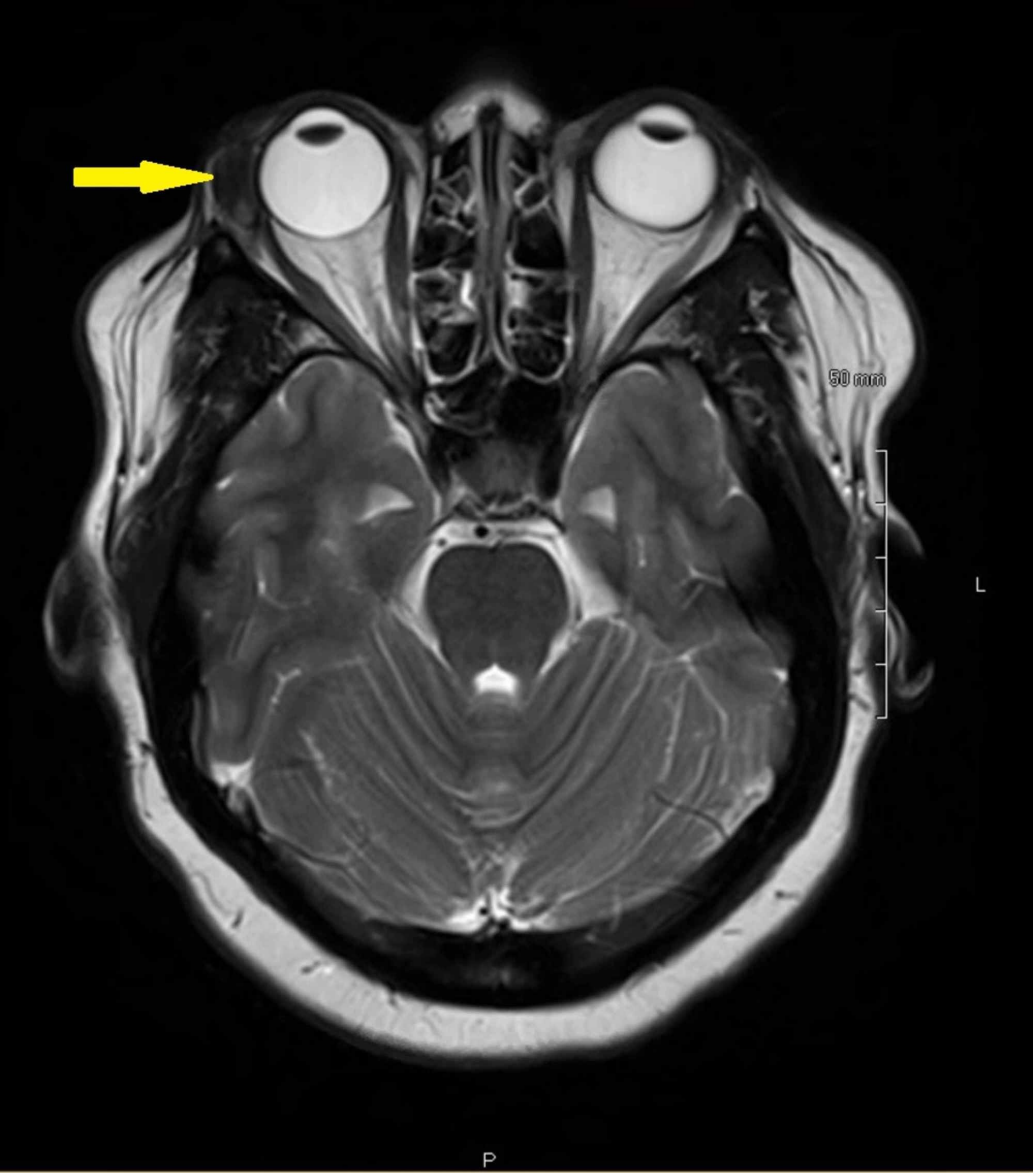
MRI brain and orbit without contrast MRI brain and orbit without contrast. T2 axial sequence showing a 3.1 x 1.6 cm hypointense lesion involving the right lacrimal gland.

A 42-year-old female was referred for evaluation of a painless right orbital mass and upper lid ptosis that has been worsening over the past year. She denied any pain or vision changes. Her past medical history was significant only for hypertension controlled with hydrochlorothiazide and diltiazem. Upon presentation, her best corrected visual acuity was 20/20 OU, pupils were equal round and reactive without afferent pupillary defect (APD), and extraocular movements were full. Significant findings included a mechanical ptosis of the right upper eyelid (Figure 1). Margin reflex distance 1 (MRD1) was 0.5 mm OD and 2 mm OS, palpebral fissures measured 10 mm OD and 12 mm OS, and Hertel exophthalmometry measured base 100 with 23 mm OD and 20 mm OS. All other exam findings were normal. MRI of the brain and orbit without contrast showed a supertemporal right orbital mass involving the lacrimal gland (Figure 2). Orbitotomy of the right eye with an excisional biopsy of the mass was performed and sent for histopathologic evaluation.

**Figure 3:**
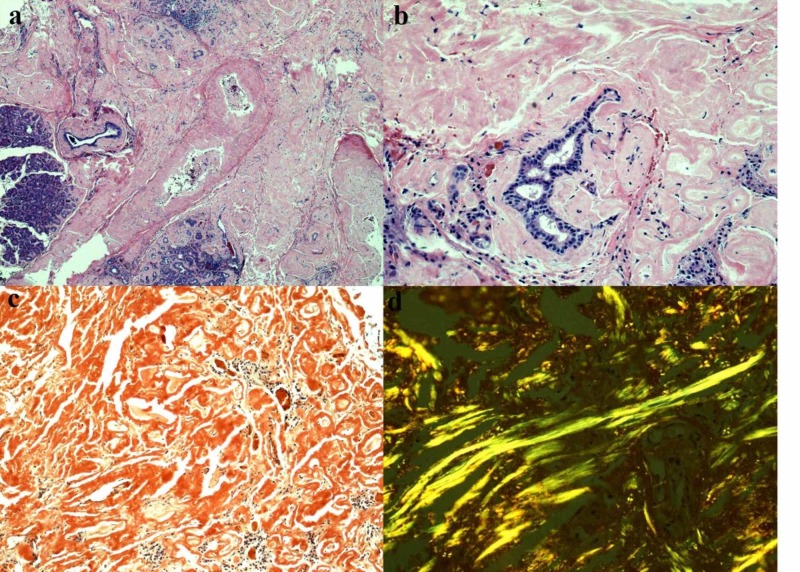
Histopathology slides of biopsy specimen (a & b) Lobules of lacrimal gland acini extensively replaced by amorphous, pale eosinophilic deposits with retention of some intralobular and interlobular ducts. (c) Typical “brick-red” hue of amyloid with Congo Red. (d) Amyloid painted “apple-green” by Congo Red histochemical stain as visualized by polarized light microscopy.

Histopathological examination of the 2.0 x 1.5 x 0.5 cm specimen revealed effaced lobules of lacrimal gland acini invested by amorphous and acellular deposits of pale, eosinophilic material (Figure 3a, 3b) tinted brick-red by Congo Red histochemical stain (Figure 3c). Under polarized light microscopy, these deposits displayed “apple-green” birefringence characteristic of amyloid (Figure 3d). A few intralobular and interlobular ducts persisted along with a few remaining lobules that contained a plasma cell infiltrate; in situ hybridization (ISH) for Kappa and Lambda failed to document light chain restriction (not shown).

The differential diagnosis included primary isolated amyloidosis of the lacrimal gland and multiple myeloma. The patient was referred to hematology/oncology for further workup. Hematologic and urine studies including anti-nuclear antibody, rheumatoid factor, anti-cyclic citrullinated peptide antibody, serum electrophoresis, immunofixation, quantitative immunoglobulins and free light chain assay were all normal, making lacrimal gland primary isolated amyloidosis the leading diagnosis.

Subsequently, the patient underwent uncomplicated levator advancement of the right upper eyelid for correction of her ptosis.

## Discussion

Amyloidosis of the lacrimal gland is a benign localized inflammatory process with only two of 19 reported cases showing systemic involvement [3,4,8]. Primary localized amyloidosis is typically formed from immunoglobulin light chain that is deposited by clonal B lymphocyte populations [9]. The presence of lymphatic ducts in the lacrimal gland may explain its involvement in primary localized orbital amyloidosis [1,8,9].

Eighty-one percent of reported cases of primary amyloidosis of the lacrimal gland have been in females with the majority of patients presenting in the sixth decade of life [3]. The most common clinical signs of amyloidosis at presentation include a visible periocular mass (94%) and ptosis (44%) [3]. Periorbital discomfort and limitations of ocular motility are the most frequently encountered symptoms at presentation [1,3,9-11].

While not diagnostic, imaging with CT or MRI is recommended in all cases of suspected ocular adnexal amyloidosis [1,3]. In lacrimal gland disease computed tomography (CT) will show a mass in the lacrimal fossa without bony erosions [12]. While there are a lack of well-established findings in the literature, MRI may show iso-intense enlarged lacrimal gland on T1 imaging and a hypo-intense gland on T2 imaging with or without rim enhancement [3]. Given that this is only the third case of lacrimal gland amyloidosis where MRI results have been reported, more data is needed to better characterize the typical finding in these patients [3,6].

Definitive diagnosis of amyloidosis is dependent on histochemical analysis and a tissue biopsy that demonstrates the pathognomonic green birefringence in unidirectional polarized light following Congo red staining [2,13]. Referral to hematology/oncology for hematologic and urine testing is necessary to rule out neoplasm and systemic organ involvement [2]. On histopathological analysis, additional tests such as immunohistochemical (IHC) staining, mass spectrometry or ISH can be performed to assess presence of amyloid protein and for kappa or lambda restriction of plasma cells to characterize the nature of the amyloid [14]. While all methods of analysis can be performed for kappa and lambda chain expression, ISH can detect lower levels of mRNA than can currently be visualized with IHC making it a highly accurate method to use for light chain expression [14,15]. In the four previous cases where IHC for light chain restriction was performed, all but one indicated light chain restriction. Our case is unique in that ISH failed to show light chain restriction, making it the second reported case without light chain restriction and first to utilize more specific ISH testing [4-7]. While uncommon in cases of lacrimal gland disease, inability to demonstrate light chain restriction and heavy chain expression is common in most other types of primary isolated amyloidosis [2]. It is unclear if primary lacrimal gland amyloidosis without light chain restriction shares a common etiology and prognosis of disease displaying these characteristics. Surgical excision is the preferred treatment modality for this condition [3,9]. There are no clear guidelines for radiotherapy for lacrimal gland amyloidosis and the risks thereof should be considered. Most cases report adjuvant external beam radiotherapy for refractory disease or that with extraocular muscle involvement [3,9,16].

## Conclusions

Primary isolated amyloidosis of the lacrimal gland is a rare disease entity that is still being characterized on a case-by-case basis. It is essential to report the key histopathological findings when reporting this disease, as it is unclear if disease with and without light chain restriction shares a common etiology and prognosis. Better characterization of MRI findings may help to improve detection of this disease entity. Lacrimal gland amyloidosis should be considered in the differential diagnosis when a patient presents with a slow growing, firm mass noted near the lacrimal fossa.
